# The Role of Palmitoylation in Signalling, Cellular Trafficking and Plasma Membrane Localization of Protease-Activated Receptor-2

**DOI:** 10.1371/journal.pone.0028018

**Published:** 2011-11-29

**Authors:** Mark N. Adams, Melinda E. Christensen, Yaowu He, Nigel J. Waterhouse, John D. Hooper

**Affiliations:** 1 Mater Medical Research Institute, Aubigny Place, Raymond Terrace, South Brisbane, Queensland, Australia; 2 Institute of Health and Biomedical Innovation, Queensland University of Technology, Kelvin Grove, Queensland, Australia; University Paris Sud, France

## Abstract

Protease-activated receptor-2 (PAR2) is a G protein coupled receptor (GPCR) activated by proteolytic cleavage of its amino terminal domain by trypsin-like serine proteases. This irreversible activation mechanism leads to rapid receptor desensitization by internalisation and degradation. We have explored the role of palmitoylation, the post-translational addition of palmitate, in PAR2 signalling, trafficking, cell surface expression and desensitization. Experiments using the palmitoylation inhibitor 2-bromopalmitate indicated that palmitate addition is important in trafficking of PAR2 endogenously expressed by prostate cancer cell lines. This was supported by palmitate labelling using two approaches, which showed that PAR2 stably expressed by CHO-K1 cells is palmitoylated and that palmitoylation occurs on cysteine 361. Palmitoylation is required for optimal PAR2 signalling as Ca^2+^ flux assays indicated that in response to trypsin agonism, palmitoylation deficient PAR2 is ∼9 fold less potent than wildtype receptor with a reduction of about 33% in the maximum signal induced via the mutant receptor. Confocal microscopy, flow cytometry and cell surface biotinylation analyses demonstrated that palmitoylation is required for efficient cell surface expression of PAR2. We also show that receptor palmitoylation occurs within the Golgi apparatus and is required for efficient agonist-induced rab11a-mediated trafficking of PAR2 to the cell surface. Palmitoylation is also required for receptor desensitization, as agonist-induced β-arrestin recruitment and receptor endocytosis and degradation were markedly reduced in CHO-PAR2-C361A cells compared with CHO-PAR2 cells. These data provide new insights on the life cycle of PAR2 and demonstrate that palmitoylation is critical for efficient signalling, trafficking, cell surface localization and degradation of this receptor.

## Introduction

Protease-activated receptors (PARs) are a subfamily of class A G protein-coupled receptors (GPCRs) consisting of four members, designated PAR1-4. Unlike other GPCRs which are activated by reversible binding of soluble ligand, these receptors are irreversibly activated by proteases; almost exclusively members of the trypsin-fold serine protease family. Proteolytic cleavage within the PAR extracellular amino terminal domain exposes a new amino terminus, or tethered ligand, which binds intramolecularly to induce intracellular signal transduction [1], [2].

The second PAR discovered, PAR2, is widely expressed and is thought to contribute to a range of normal and disease processes including embryogenesis, pain and nociception, acute and chronic inflammation, arthritis and cancer [3], [4], [5], [6], [7], [8]. PAR2 is activated by numerous trypsin-like serine proteases including trypsin, mast cell tryptase, tissue factor complexed with factor VIIa and factor Xa, and kallikrein 4, 5, 6 and 14 [9], [10], [11], [12], [13]. As for PAR1 and PAR4, PAR2 can also be activated by hexapeptides, termed agonist peptides (AP), that mimic the tethered ligand. Cell surface expression of PAR2 enables the cell to respond normally or aberrantly to protease challenge by inducing signal transduction via coupled hetrotrimeric G protein subunits Gα_q_, Gα_i_ and Gα_12/13_ to elicit mitogen-activated protein kinase (MAPK) signalling, calcium mobilisation, Rho and Rac activation and stimulation of NF-κB and gene transcription [1]. PAR2 also signals independent of G proteins via β-arrestin mediated activation of the MAPK pathway [14].

Due to the irreversible nature of PAR2 activation, rapid mechanisms are required to prevent sustained and excessive receptor signalling. Following proteolysis, PAR2 is phosphorylated within the carboxyl terminus and ubiquitinated on intracellular lysine residues before interacting with β-arrestins enabling receptor desensitisation and internalisation [15], [16]. PAR2 is trafficked via the early and late endosomes and degraded within lysosomes [17], [18]. A consequence of irreversible activation and rapid desensitisation and degradation, is that large intracellular PAR2 stores are required to rapidly replenish the cell surface with nascent receptors thereby re-establishing the ability of cells to sense proteolytic activity. Although the mechanisms controlling this process are poorly characterised, it is clear that the GTPase rab11a participates in intracellular trafficking of PAR2 within the Golgi apparatus and toward the plasma membrane [19].

Post translational modifications such as glycosylation, phosphorylation and ubiquitination of PAR2 are critical regulators of PAR2 function [15], [16], [20]. Recently, Botham and colleagues have also shown that PAR2 is modified by the post-translation addition of palmitate to cysteine 361 (C361) [21]. Palmitoylation is often dynamic and reversible and occurs commonly for GPCRs on one or more carboxyl terminal cysteines found 10 to 14 residues following the seventh transmembrane domain [22]. In addition to these thioester linkages (so called *S*-palmitoylation), there are a small number of examples where palmitate addition to cysteine is followed by structural rearrangement leading to palmitate modification of an amide (*N*-palmitoylation) [23]. Palmitoylation anchors the GPCR carboxyl domain to the inner leaflet of cell membranes resulting in the formation of an intracellular eighth α-helix (H8) and has diverse effects on GPCR function including regulation of cellular trafficking [24], [25]. It is also proposed to regulate GPCR signalling by modifying the conformation of the carboxyl terminus thereby impacting on G protein coupling [26], [27], [28].

Here we report on the role of palmitoylation in regulating cell surface expression of endogenous PAR2 and use stably and transiently expressing cells to explore the function of this modification in receptor trafficking, signalling and degradation. In contrast with Botham and co-workers, who show that palmitoylation deficient PAR2 stably expressed by CHO-Pro5 cells is more highly expressed on the cell surface than wildtype receptor [21], we demonstrate that palmitoylation of PAR2 is required for efficient plasma membrane receptor expression in 3 different endogenously expressing cell lines. Our data from these cell lines are supported by detailed studies comparing CHO-K1 cells stably expressing wildtype or C361A PAR2. We also explore the role of receptor agonism in PAR2 palmitoylation and how this post-translational modification regulates rab11a trafficking to the cell surface, agonist induced β-arrestin relocation to the cell surface and receptor degradation. Our data suggest that *S*-palmitoylation is required for optimal PAR2 signalling, cellular trafficking and plasma membrane localization.

## Methods

### Antibodies and reagents

Anti-PAR2 antibody N19 was from Santa Cruz Biotechnology (Quantum Scientific Pty Ltd, Murarrie, Australia) and an anti-myc epitope (EQKLISEEDL) antibody was from Cell Signaling Technology (Genesearch Pty Ltd, Arundel, Australia). An anti-GAPDH antibody was from Sigma Aldrich (Castle Hill, Australia). Alexa Fluor 680-conjugated streptavidin and Alexa Fluor 488-, Alexa Fluor 647- and Alexa Fluor 680-conjugated secondaries were from Invitrogen (Mulgrave, Australia) and IRdye 800-conjugated secondary antibodies were from LiCor (Millennium Science, Surrey Hills, Australia). PAR2 activating peptide (AP; SLIGRL-NH_2_) was purchased from Auspep (Parkville, Australia). Active bovine trypsin was obtained from Worthington Biochemical (Scimar Pty Ltd, Templestowe, Australia). *N*-glycosidase F was purchased from New England Biolabs (Genesearch Pty Ltd). Brefeldin A, 2-bromopalmitate, cerulenin, CuSO_4_ III, cycloheximide, hydroxylamine, MG132, monensin, N-ethylmaleimide (NEM), nocodazole, palmitate, sodium fluoride, sodium vanadate, t-butanol, tris[(1-benzyl-1H-1,2,3-triazol-4-yl)methyl] amine (TBTA) and tris(2-carboxyethyl)phosphine (TCEP) were from Sigma Aldrich. 17-Octadecynoic acid (17-ODYA) was from Cayman Chemicals (Sapphire Bioscience, Waterloo, Australia) and biotin-azide and Fura-2 were purchased from Invitrogen. EZ-link NHS-SS-biotin and EZ-link HPDP-biotin were from Pierce (Thermo Fisher Scientific, Scoresby, Australia). Protease inhibitor cocktail was purchased from Roche (Castle Hill, Australia). Live cell imaging 8 chamber μ-slides were obtained from Ibidi (In Vitro Technologies Pty Ltd, Noble Park, Australia). Protein concentrations were determined using a bicinchoninic acid assay (BCA) kit from Pierce.

### Expression constructs and mutagenesis

The human PAR2 open reading frame incorporating 3′ sequence encoding a carboxyl terminal myc epitope (sequence) was amplified by PCR using Expand High Fidelity polymerase mixture (Roche) from a previously described construct [12] and cloned into the pIRESneo2 vector (Clonetech, Scientifix Pty Ltd, Clayton, Australia). Site-directed mutagenesis, to mutate this PAR2-myc construct at cysteine 361 to alanine (C361A) was performed using *Pfu* Ultra polymerase (Agilent Technologies, Forest Hill, Australia). A construct encoding PAR2 tagged at the carboxyl terminal with green fluorescent protein (GFP) was described previously [12]. An expression construct encoding PAR2 with a carboxyl terminal monomeric Cherry (mCherry) was generated from the PAR2-GFP construct. The GFP encoding fragment was removed from PAR2-GFP by restriction digestion using BamHI and NotI (New England Biolabs) and this was replaced with mCherry encoding DNA [29]. The sequence of all constructs was confirmed by DNA sequencing at the Australian Genome Research Facility (St. Lucia, Australia). Expression constructs encoding amino terminal GFP tagged wildtype Rab11a and dominant negative mutant Rab11a-S25N were from Dr Marci Scidmore (Cornell University) [30]. Expression constructs encoding carboxyl terminal GFP tagged β-arrestin-1 & -2 were from Dr Robert Lefkowitz (Duke University Medical Center) [31].

### Cell culture and transfections

All cell culture media and reagents were from Invitrogen except for fetal calf serum which was from Sigma Aldrich. Cells were purchased from ATCC (Manassas, VA). Chinese hamster CHO-K1 (CHO) cells were grown in DMEM and prostate cancer lines PC3, DU145 and 22Rv1 were grown in RPMI1640 medium, supplemented with 10% fetal calf serum (FCS), 100 units/ml of penicillin, and 100 units/ml of streptomycin in a 5% CO_2_ humidified atmosphere at 37°C. All cells were passaged using 0.5 mM EDTA in PBS. Transfections were performed using either Lipofectamine 2000 or Lipofectamine LTX (Invitrogen). For stable transfections CHO cells were transfected with pIRESneo2 (vector), PAR2-myc or PAR2-C361A-myc using Lipofectamine 2000 then selected in G418 (800 µg/ml) containing media for 10 days before clones were selected for expansion and characterisation.

### Cell membrane preparation

Crude cell membrane extracts were collected as previously described [32] with some alterations. Briefly, cells at 50% confluence were washed with PBS and distilled H_2_O for 30 sec to induce hyptonic cell shock. Swollen cells were mechanically resuspended in membrane buffer (5 mM Tris, pH 7.5, 0.5 mM EDTA, 1× protease inhibitor cocktail, 1 mM sodium vanadate and 10 mM sodium fluoride) and disrupted by several passes through a 26 gauge needle. Cellular debris was removed by centrifugation (800 g for 10 min at 4°C). Crude membrane preparations were collected from the supernatant by ultracentrifugation (100,000 g for 1 h at 4°C) then resuspended in lysis buffer containing 50 mM Tris pH 7.4, 150 mM NaCl, 5 mM EDTA, 1% Triton X-100 (v/v) and 1× protease inhibitor cocktail (Roche) before protein quantification using a BCA kit (Pierce).

### Analysis of palmitoylation by acyl-biotinyl exchange chemistry

PAR2 palmitoylation was assessed by an acyl-biotinyl exchange approach (ABE; [33]) described previously [34]. Crude membrane extracts, isolated as above, were prepared from cells at 50% confluence. To assess the effect of PAR2 agonism on palmitoylation, before isolation of cell membranes cells were treated with PAR2 AP (100 µM). To prevent protein palmitoylation, cells were incubated with 2-bromopalmitate for 16 h prior to collection of crude membrane. Membrane pellets were solubilised for 1 h at 4°C in lysis buffer containing 50 mM HEPES pH 7.4, 150 mM NaCl, 5 mM EDTA, 1% Triton X-100 (v/v), 1× protease inhibitor cocktail (Roche) and, to block free thiols, 10 mM NEM. Solubilized protein (100 µg) was chloroform-methanol (1∶3∶2 protein∶methanol∶chloroform; CM) precipitated and protein pellets were resuspended in a buffer containing 4% SDS (4SB; 4% SDS, 50 mM HEPES pH 7.4, 5 mM EDTA) and diluted five fold with lysis buffer containing 10 mM NEM and incubated at 4°C overnight with gentle agitation. Excess NEM was removed by three sequential CM precipitations followed by resuspension in 4SB (50 µl). Samples were divided into two equal portions. To cleave thioester bonds and allow incorporation of a biotin moiety at exposed sulphur atoms, one portion was diluted five fold in buffer (0.2% Triton X-100, 1× protease inhibitor cocktail) containing hydroxylamine (0.7 M) and EZ-link HPDP-biotin (1 mM). As a control the other portion was diluted five fold in the same buffer in which hydroxylamine was replaced with Tris (50 mM Tris pH 7.4). In the absence of hydroxylamine, palmitate groups are not removed thereby preventing biotinylation mediated purification. Each portion was incubated at ambient temperature for 1 h with gentle agitation. Unreacted biotin was removed by three sequential CM precipitations and the protein pellet was resuspended in 4SB and diluted 10 fold in Tris containing lysis buffer. Biotinylated proteins were affinity purified using streptavidin beads (Pierce) by incubation at 4°C for 1 h. Bound proteins were eluted from washed beads with SDS sample buffer and subjected to denaturing polyacrylamide gel electrophoresis (SDS-PAGE). Palmitoylated PAR2 was detected by anti-myc Western blot analysis.

### Analysis of palmitoylation by metabolic labelling with an alkyne containing palmitate analogue followed by *in vitro* copper catalysed alkyne-azide cycloaddition (click) chemistry

PAR2 palmitoylation was also examined using a modified version of a recently described method using an alkyne containing palmitate analog (17-ODYA) reacted to biotin-azide via click chemistry [35]. To inhibit endogenous palmitate synthesis cells were preincubated for 1 h at 37°C with cerulenin (5 µg/mL), before metabolic labelling with the palmitate analog 17-ODYA (25 µM) for 2 h at 37°C. To assess the effect of PAR2 agonism on palmitoylation, cells were labelled with 17-ODYA in the presence or absence of PAR2 AP (100 µM). To examine PAR2 palmitoylation in particular organelles, cells were labelled with 17-ODYA for 2 h in the presence of protein trafficking and organelle function inhibitors: Brefeldin A (5 µg/mL; blocks ER-to-Golgi protein traffic [36]), nocodazole (20 µg/mL; blocks ER-to-pre-Golgi protein traffic [37]), monensin (10 µM; blocks medial-Golgi-to-post-Golgi traffic [36]), and 2-bromopalmitate (100 µM; inhibits protein palmitoylation [38]). Nocodazole was added to cells 15 min before labelling with 17-ODYA, while Brefeldin A, monensin and MG132 were added 30 min prior to labelling with 17-ODYA. 2-Bromopalmitate was incubated with cells for 16 h before collection of membrane fractions. Crude membrane fractions, isolated as described above, were CM precipitated then resuspended in PBS containing 1.2% SDS by sonication and heating at 70°C for 10 min. Proteins (100 µg at 1 mg/mL) labelled with alkyne containing 17-ODYA were reacted with biotin-azide via standard click chemistry conditions; involving the addition of 100 µM biotin-azide (1 mM stock dissolved in DMSO), 1 mM TCEP (50 mM stock dissolved in H_2_O), 100 µM TBTA (1.7 mM stock dissolved in 20% DMSO/80% t-butanol) and 1 mM CuSO_4_ (50 mM stock dissolved in PBS) in order, followed by incubation in the dark at ambient temperature for 1 h. Samples were CM precipitated to remove excess biotin, resuspended in PBS containing 0.8% SDS and diluted eight fold in lysis buffer. Palmitoylated proteins modified with a biotin moiety were affinity purified using streptavidin beads by incubation at 4°C for 1 h with gentle agitation. Bound proteins were eluted from washed beads with SDS sample buffer and subjected to SDS-PAGE followed by anti-myc Western blot analysis to detect palmitoylated PAR2. In experiments to assess whether palmitoylation was found exclusively on cysteines and not at amids, samples were boiled in 0.7 M hydroxylamine (to cleave thioester bonds) or 50 mM Tris pH 7.4 (control) prior to immobilisation on streptavidin beads.

### Cell surface biotinylation

Cell surface proteins were isolated using cell impermeant EZ-link NHS-SS-biotin (1.22 mg/mL) as described previously [39]. Briefly, cells at 50% confluence stably transfected with PAR2-myc, PAR2-C361A-myc or vector were washed with PBS and biotinylated for 1 h at 4°C. Cells were washed with PBS and whole cell lysates collected in lysis buffer (1% (v/v) Triton X-100, 50 mM Tris/HCl (pH 7.4), 150 mM NaCl and 1× protease inhibitor cocktail). After removal of cellular debris by centrifugation (3000 rpm for 10 min at 4°C), lysates were incubated with streptavidin beads (Pierce) for 30 min at 4°C with gentle agitation. Biotinylated cell surface proteins immobilised on streptavidin beads were pelleted by centrifugation (3000 rpm for 5 min at 4°C) and together with intracellular proteins present in the supernatant were examined by Western blot analysis.

### Receptor endocytosis

PAR2 endocytosis was assessed as previously described [40]. Briefly, cell surface proteins were labelled with cell impermeant EZ-link NHS-SS-biotin (0.5 mg/mL) for 30 min at 4°C, then washed before incubation at 37°C in culture media containing PAR2 AP (100 µM) and MG132 (20 µM) for 5, 15 and 30 min to allow receptor internalisation. Surface biotin was removed by incubating cells at 4°C in reducing buffer (50 mM 2-mercaptoethanesulfonic acid sodium salt (MeSNA), 100 mM Tris pH 8.6, 100 mM NaCl) for 30 min before excess MeSNA was quenched with 60 mM iodoacetamide for 15 min in PBS. Whole cell lysates were collected in lysis buffer and incubated with streptavidin beads to isolate internalised PAR2 which was examined by Western blot analysis.

### Western blot analysis

Whole cell lysates collected in lysis buffer, cell membrane preparations, proteins eluted from streptavidin beads, and proteins collected from the cell surface biotinylation protocol were separated by SDS/PAGE and transferred to nitrocellulose membranes. After blocking with Odyssey blocking buffer (LiCor), membranes were incubated with primary antibodies overnight at 4°C, washed and then incubated with species-appropriate AlexaFluor 680 or IRdye 800-conjugated secondary antibodies for 45 min at ambient temperature. Following washing, membranes were scanned on an Odyssey infrared imaging system (LiCor). Consistent protein loading and transfer was determined by reprobing membranes with either an anti-GAPDH antibody or AlexaFluor-680-conjugated streptavidin. Where relevant, signal intensity was determined by densitometry analysis using Odyssey software (LiCor).

### Flow cytometry

Adherent cells at 50% confluence were lifted non-enzymatically, counted and 2.5×10^5^ cells were washed and stained with goat anti-PAR2 antibody N19 (2 µg/1×10^6^ cells) in PBS containing 2% FCS for 30 min at 4°C. After washing with PBS, cells were stained with an AlexaFluor 488-conjugated secondary antibody (Invitrogen) and 20,000 events were collected and analysed on a Beckman Coulter FC500 flow cytometer. For cell surface repopulation experiments, resuspended cells treated with 50 nM trypsin for 15 min at 37°C were washed then incubated in trypsin-free DMEM for 0, 15 and 30 min and placed on ice, followed by staining with anti-PAR2 N19 antibody. For cells expressing GFP, a donkey anti-goat Alexa Fluor 647-conjugated secondary antibody was used to prevent spectral overlap and cells were analysed on a Beckman Coulter CyAN flow cytometer. For all experiments, mean fluorescence intensity (MFI) values were calculated by subtracting secondary only staining from specific anti-PAR2 staining.

### Calcium mobilisation assay

Analysis of calcium mobilisation was performed as previously described [12]. Cells at 50% confluence lifted nonenzymatically were loaded with the fluorescence indicator Fura-2 acetoxymethyl ester (1 µM; Invitrogen) for 1 h at 37°C in buffer containing 25 mM HEPES pH 7.4, 121 mM NaCl, 5.4 mM KCl, 0.8 mM MgCl_2_, 1.8 mM CaCl_2_, 5.5 mM glucose, 2.5 mM probenecid and 0.01% (v/v) pluronic acid. Cells were washed with PBS and resuspended in the same buffer lacking Fura-2 and pluoronic acid. A range of 0.1–1000 nM trypsin was used for dose-response experiments. Calcium mobilisation was monitored using a Polarstar Optima fluorescent plate reader (BMG Labtech Pty Ltd, Mornington, Australia). All experiments were performed in triplicate on three independent occasions with results displayed as mean ± SD normalised to maximal PAR2 response.

### Live cell Confocal microscopy

Cells seeded in 8 chamber μ-slides (Ibidi) were transiently co-transfected with GFP and mCherry containing expression constructs for 12 h and imaged at 37°C in a 5% CO_2_ atmosphere using a Zeiss LSM510 confocal microscope. Images were processed using MetaMorph software and displayed using Corel Draw X5.

### Statistics

Results are mean ± SD of at least 3 independent experiments. For experiments assessing rab11a mediated trafficking of PAR2 and degradation of PAR2, statistical significance was assessed using a 2-way ANOVA test. All other experiments were assessed by Student's t test. *P*<0.05 was considered significant.

## Results

### Inhibition of palmitoylation reduces cell surface expression of endogenous PAR2

**Figure 1:**
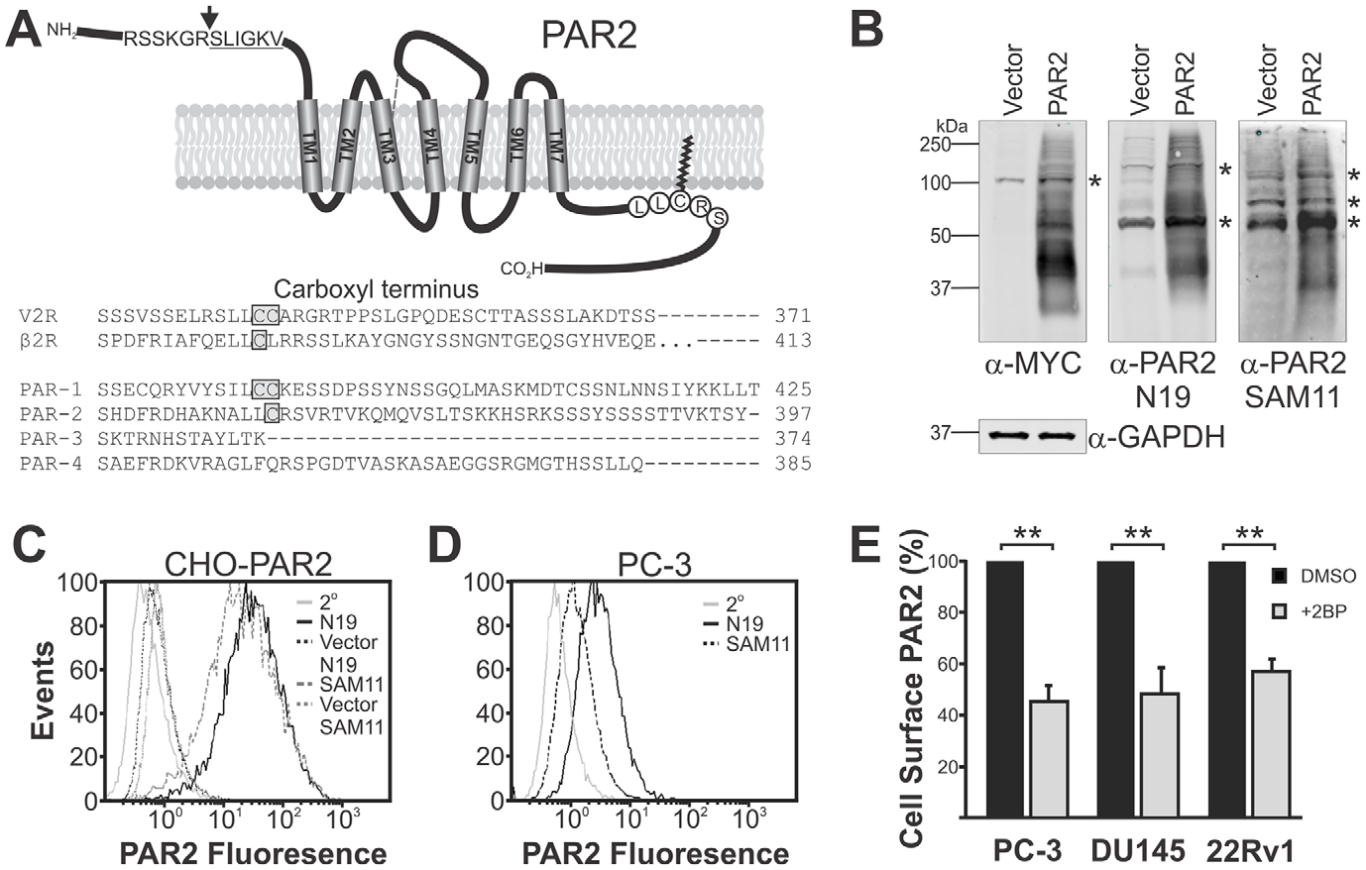
Inhibition of palmitoylation reduces cell surface expression of endogenous PAR2. *A*, Upper panel; schematic representation of the structure of human PAR2 including the tethered ligand sequence (underline) within the amino terminal domain, C361 within a consensus palmitoylation motif present in carboxyl terminal domain, seven transmembrane (TM1 to TM7) domains, and a disulfide bond linking TM3 and the second extracellular loop (dotted line). Lower panel; protein sequence alignment of seventh transmembrane and carboxyl terminal residues of arginine vasopressin receptor-2 (V2R; NM_000054; residues 329–371), β2-adrenergic receptor (β2R; NM_000024.5; residues 329–413), PAR1 (NM_001992.3; residues 375–425), PAR2 (NM_005242.4; residues 347–397), PAR3 (NM_004101.2; residues 362–374) and PAR4 (NM_003950.2; residues 344–385). The known palmitoylation sites of V2R and β2R and the consensus palmitoylation sites of PAR1 and PAR2 are boxed. *B*, Western blot analysis of lysates from CHO cells stably transfected with vector or PAR2-myc probed with anti-PAR2 antibodies N19 and SAM11 and anti-myc and anti-GAPDH antibodies. *, non-specific band. *C*, Anti-PAR2 flow cytometry analysis of non-permeabilised CHO cells stably transfected with vector or PAR2-myc using antibodies N19 and SAM11. MFI values: CHO-PAR2 cells with N19 51.9±2.5; CHO-PAR2 cells with SAM11 41.6±3.2; signal from incubation of CHO-PAR2 cells with secondary antibody (2°), CHO-vector with antibody N19 (Vector N19) and CHO-vector with antibody SAM11 (Vector SAM11) were below 10^0^. *D*, Flow cytometry analysis of cell surface PAR2 endogenously expressed by non-permeabilised PC-3 cells using anti-PAR2 N19 and SAM11 antibodies. MFI values: N19 4.3±0.5; SAM11 2.3±0.9; secondary antibody (2°) 0.60±0.06. *E*, Graphical representation of the effect of blocking palmitoylation on cell surface expression of PAR2. Plasma membrane levels of PAR2, expressed endogenously by PC3, DU145 and 22Rv1 cells, were determined by flow cytometry using the anti-PAR2 N19 antibody. Non-permebilised cells were either treated with DMSO (negative control) or 2-bromopalmitate (+2BP) for 16 h. Secondary antibody only MFI values were subtracted from N19 values before calculation of the level of cell surface PAR2 present on 2BP treated cells relative to DMSO treated cells. Values were determined from 3 independent experiments and are shown as mean ± SD. **, *P*<0.001.

Shown in Figure 1A is an alignment of amino acids from transmembrane domain 7 to the carboxyl terminal of two known palmitoylated GPCRs, vasopressin V2 receptor (V2R) [41] and β2-adrenergic receptor (β2R) [42], and the four members of the PAR family. Recently Botham and co-workers have demonstrated that C361 of PAR2 (Fig. 1A schematic), which aligns with the palmitoylation sites of V2R (a di-cysteine) and β2R (a mono-cysteine), is the primary palmitoylation site of the GPCR PAR2 in over-expressing CHO-Pro5 cells [21]. For the purpose of examining the role of palmitoylation in PAR2 cell surface localization in endogenous expressing cells, we first examined the ability of anti-PAR2 antibodies, SAM11 and N19, to detect PAR2. As shown in Figure 1B, Western blot analysis showed that both antibodies detect PAR2 as a smear from ∼35 to ∼150 kDa in lysates from stably expressing CHO cells. However, non-specific signals were also apparent with both antibodies (Fig. 1B, vector lanes) indicating that these would not be ideal for Western blot based approaches to evaluate PAR2 palmitoylation. In contrast, flow cytometry analysis indicated that both antibodies specifically detect PAR2 expressed on the surface of stably expressing CHO cells with no evidence of non-specific binding apparent from staining of CHO-vector cells (Fig. 1C). Flow cytometry analysis of prostate cancer PC-3 cells, that are known to endogenously express PAR2 [12], indicated that both antibodies can detect the endogenous receptor (Fig. 1D). As the signal with the N19 antibody was stronger, this antibody was used in flow cytometry assays to examine the role of palmitoylation in cell surface expression of PAR2 in PC-3 cells and two other endogenous expressing prostate cancer lines, DU145 and 22Rv1. As palmitoyaltion is known to impact on cell surface localization of other GPCRs [25], we evaluated the effect of the palmitoylation inhibitor 2-bromopalmitate, on the plasma membrane location of PAR2 expressed by these three cell lines. This inhibitor functions by inhibiting the enzymes required for palmitoylation, palmitoyl acyltransferases (PATs; [38]), and as shown in Figure 1E, reduced cell surface expression of PAR2 in each cell line by ∼50%. These data suggest that endogenous PAR2 is likely palmitoylated in PC-3, DU145 and 22Rv1 cells.

### Characterisation of palmitoylation deficient PAR2 stably expressed by CHO cells

**Figure 2:**
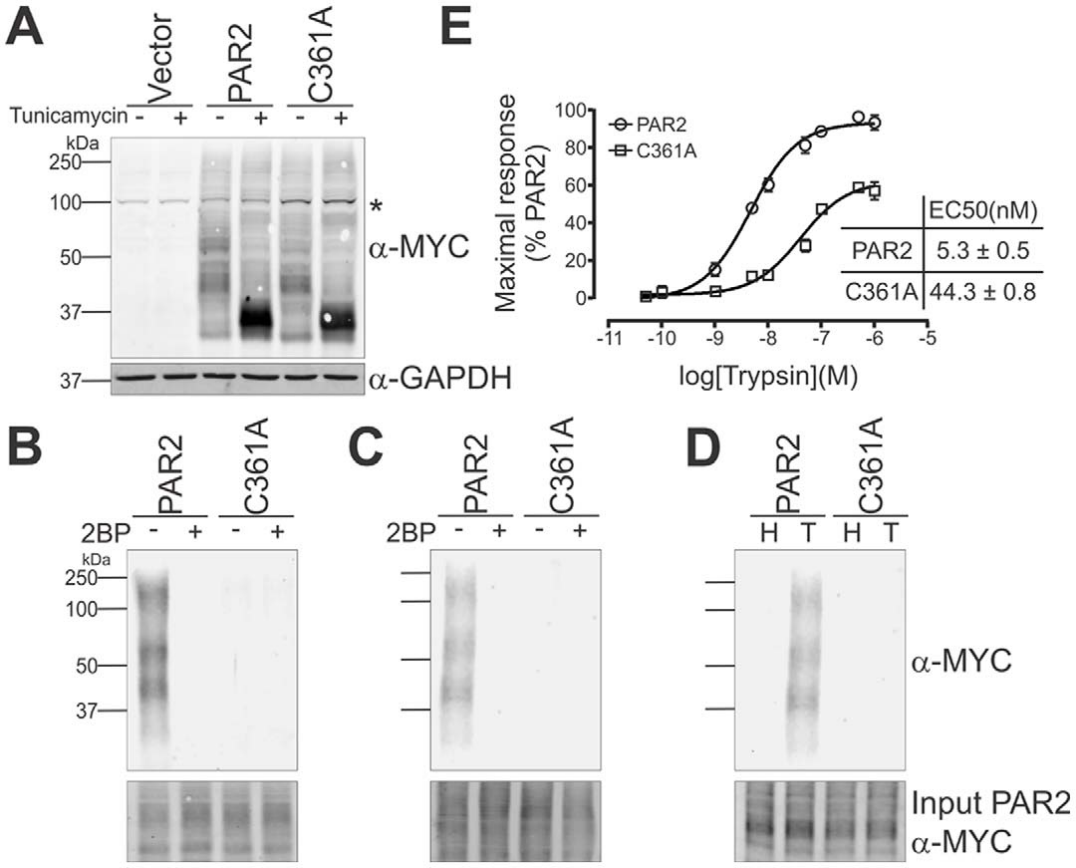
PAR2 is palmitoylated on cysteine 361. *A*, Anti-myc and anti-GAPDH Western blot analysis of lysates from CHO cells stably expressing either vector, PAR2-myc or PAR2-C361A-myc. Before collection of lysates cells were either untreated or treated with the *N*-glycosylation inhibitor tunicamycin. *, non-specific band. *B*, Analysis of PAR2 palmitoylation using acyl-biotinyl exchange chemistry. Membrane fractions were collected from CHO-PAR2-myc and CHO-PAR2-C361A-myc cells either untreated or treated with 2-bromopalmitate (2BP) for 16 h. Free thiols on membrane proteins were blocked with NEM, endogenous palmitoyl groups were removed using hydroxylamine followed by sulphydryl-reactive biotinylation of liberated cysteines. Biotinylated proteins were isolated by streptavidin beads, eluted and subjected to anti-myc Western blot analysis to examine PAR2 palmitoylation. *C*, Analysis of PAR2 palmitoylation by examining cells metabolically labelled with an alkyne containing palmitate analogue (17-ODYA) followed by click chemistry. PAR2-myc and PAR2-C361A-myc CHO cells were incubated with 2-bromopalmitate for 16 h and cerulenin (5 µg/mL) for 1 h then labelled with 17-ODYA for 4 h, before collection of membrane preparations and reaction of biotin-azide to 17-ODYA labelled proteins via click chemistry. Biotinylated proteins were isolated using streptavidin beads and PAR2 palmitoylation was assessed by anti-myc Western blot analysis from bead elutes. *D*, Analysis of whether palmitoylation of PAR2 occurs exclusively on a cysteine residue and not via an amide linkage. Membrane preparations were collected from CHO-PAR2-myc and CHO-PAR2-C361A-myc 17-ODYA labelled cells. Labelled proteins were reacted with biotin-azide via click chemistry before incubation with 0.7 M hydroxylamine (H) to cleave thioester linkages or 50 mM tris pH 7.4 (T; control). Biotinylated proteins were isolated using streptavidin beads and PAR2 palmitoylation was assessed by anti-myc Western blot analysis from bead elutes. *E*, Comparison of intracellular Ca^2+^ mobilisation mediated by trypsin activation of PAR2-myc (○, EC50 5.3±0.5 nM) and PAR2-C361A-myc (□, EC50 44.3±0.8 nM) in stably expressing CHO cells. Experiments were performed in triplicate on 3 separate occasions and values are Mean +/− SD.

To directly examine the mechanisms regulating palmitoylation of PAR2 and the role of this palmitoylation in PAR2 signalling and trafficking, we generated CHO-K1 cells stably expressing PAR2-C361A-myc. As shown in Figure 2A, anti-myc Western blot analysis showed that this mutant PAR2 is expressed at similar levels to the wildtype protein in stably expressing CHO cells. In addition, Western blot analyses of lysates collected from these cells treated with the *N*-glycosylation inhibitor tunicamycin, indicated that wildtype PAR2 and the receptor mutated at C361 each carry similar levels of N-linked glycans (Fig. 2A). These cells were used in two approaches to assess palmitoylation of PAR2 at C361. In the first approach, employing ABE chemistry, free cysteines on proteins from membrane preparations were first blocked using NEM. Cysteine-palmitoyl thioester bonds were then specifically cleaved using the weak nucleophile hydroxylamine which breaks these bonds but not amide-palmitoyl linkages [23]. Liberated cysteines were then reacted with sulfhydryl reactive EZ-link HPDP-biotin to label proteins that had originally contained palmitoyl moieties. Biotinylated proteins were isolated using streptavidin beads and the level of palmitoylated PAR2 present in this fraction was assessed by anti-myc Western blot analysis. This method assesses the gross amount of PAR2 palmitoylation. As shown in Figure 2B, PAR2-myc was detected as a smear ranging from ∼35 kDa to ∼150 kDa indicating that this protein is palmitoylated. Consistently, this Western blot analysis also showed that the palmitoylation inhibitor 2-bromopalmitate completely blocked palmitoylation of PAR2. In contrast with wildtype PAR2, the C361 mutant was not pamitoylated in the presence or absence of 2-bromopalmitate.

In the second approach, using click chemistry, PAR2-myc and PAR2-C361A-myc CHO cells were treated with an inhibitor of endogenous palmitate synthesis, cerulenin, before metabolic labelling with the alkyne containing palmitate analog 17-ODYA. Membrane preparations were collected and biotin-azide reacted to 17-ODYA labelled proteins via copper catalysed alkyne-azide cycloaddition (click) chemistry. Biotinylated proteins were isolated using streptavidin beads and PAR2 palmitoylation was detected from bead elutes by anti-myc Western blot analysis. Whereas ABE chemistry examines gross whole proteome palmitoylation, metabolic labelling of live cells with a palmitate analogue, while blocking endogenous palmitate synthesis, followed by reaction with biotin-azide via click chemistry enables identification of dynamically palmitoylated proteins [43]. As shown in Figure 2C, this approach confirmed that wildtype but not C361A PAR2 is palmitoylated and that palmitoylation of wildtype PAR2 is blocked by the palmitoylation inhibitor 2-bromopalmitate. To confirm that palmitoylation of PAR2 occurs exclusively on a cysteine residue, and not via an amide linkage that can sometimes occur [44], we treated biotinylated proteins with the weak nucleophile hydroxylamine or, as a negative control, Tris, before isolation of proteins containing biotin using streptavidin beads. In contrast with the ABE chemistry approach which is not capable of detecting amide-palmitate linkages, this modification to our click chemistry protocol permits the distinction between *S*-palmitoylation and *N*-palmitoylation. As shown in Figure 2D, hydroxylamine but not Tris treatment completely removed all palmitoylation of PAR2 indicating that this receptor is exclusively palmitoylated on a cysteine residue. Consistent with the report of Botham and colleagues [21], these data indicate that PAR2 is palmitoylated at C361. Our data also indicate that this is the exclusive site at which PAR2 is palmitoylated.

We performed Ca^2+^ mobilisation assays to examine the effect of mutation of PAR2 C361 on signal transduction initiated via cleavage of this receptor by trypsin. As shown in Figure 2E, in response to trypsin PAR2-C361-myc was ∼9 fold less potent than PAR2-myc (half-maximal response (EC50) of 44.3 versus 5.3 nM) with a reduction of about 33% in the maximum signal induced via the mutant receptor. These data are comparable with the findings of Botham *et al.* (PAR2 EC50 11.4 nM and PAR2-C361A EC50 40.8 nM) and indicate that palmitoylation of C361 is essential for efficient PAR2 signal transduction [21].

### Palmitoylation of PAR2 at C361 is required for efficient cell surface receptor localization

**Figure 3:**
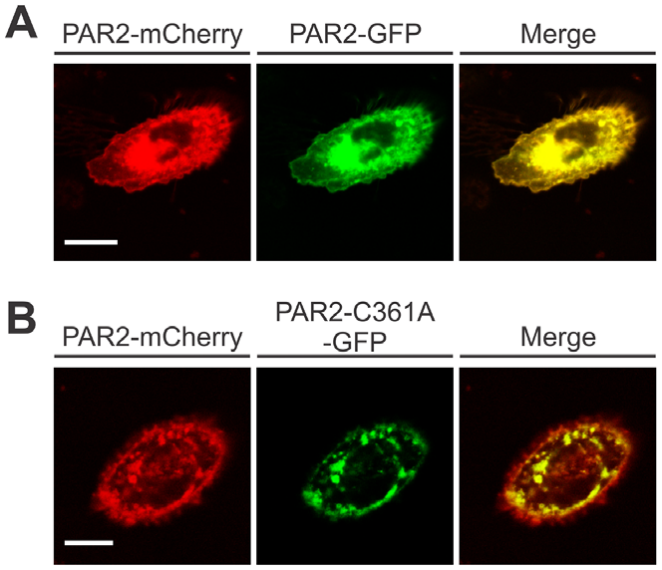
Palmitoylation of PAR2 at C361 is required for efficient cell surface receptor localization. *A*, Images of CHO cells transiently co-transfected for 12 h with PAR2-mCherry (red) and PAR2-GFP (green). The merge image shows colocalization (yellow) of these two proteins. *B*, Images of CHO cells transiently co-transfected for 12 h with PAR2-mCherry and PAR2-C361A-GFP. The merge image shows colocalization (yellow) of these two proteins and regions where only PAR2-mCherry is expressed (red). Cells were analysed using a Zeiss LSM510 confocal microscope (63× oil immersion objective lens) and images were processed using MetaMorph software and displayed using CorelDraw X5. Images of cells are representative of three independent experiments. *Scale bar*, 10 µM.

**Figure 4:**
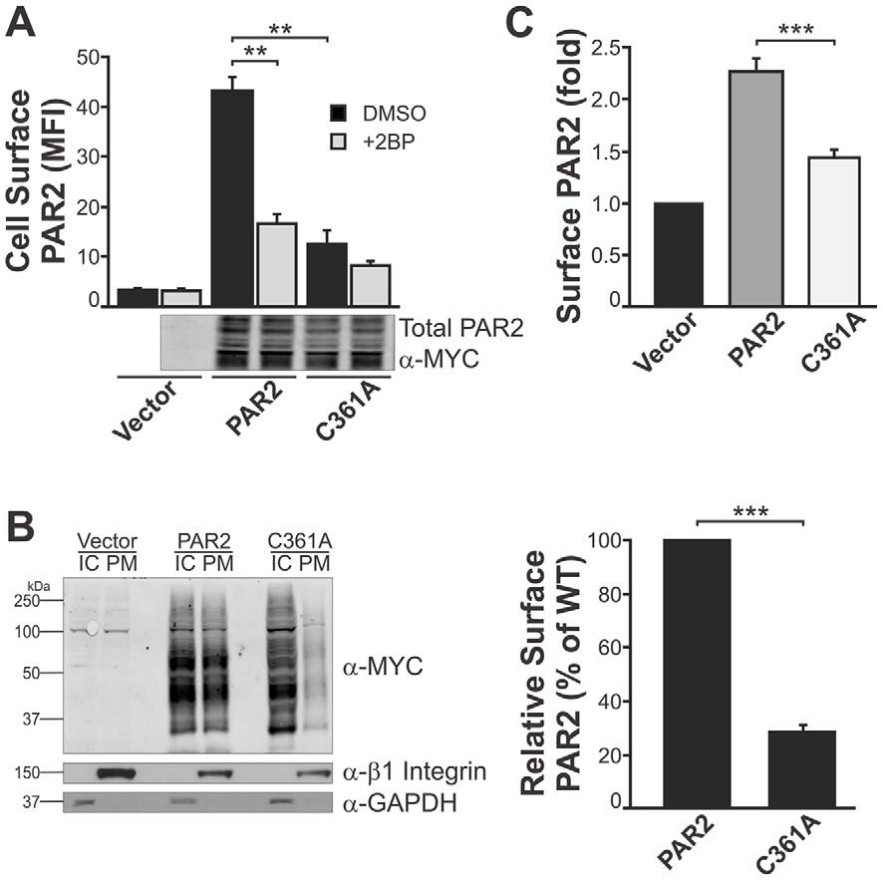
Quantitative analysis of the effect of palmitoylation on cell surface expression of PAR2. *A*, Anti-PAR2 N19 antibody flow cytometry analysis of non-permeabilised CHO-PAR2-myc and CHO-PAR2-C361A-myc cells treated with either DMSO (negative control) or 2BP for 16 h. Secondary antibody only MFI values were subtracted from N19 values and these are shown relative to values from CHO-vector untreated cells. Values were determined from 3 independent experiments and are shown as Mean ± SD. *B*, (Left panel) Examination of plasma membranePAR2 levels by cell surface biotinylation. Live cells were reacted with membrane impermeant EZ-link NHS-SS-biotin. Plasma membrane (PM) and intracellular (IC) fractions were collected from whole cell lysates and subjected to anti-myc, anti-GAPDH (control for IC fraction) and anti-β1 integrin (control for PM fraction) Western blot analysis. The data are representative of 3 independent experiments. (Right panel) Graphical representation of densitometric analysis of data from these 3 experiments. Values of PM PAR2 were normalised to β1 integrin signal and are displayed as Mean ± SD. *C*, Anti-PAR2 N19 antibody flow cytometry analysis of DU145 cells transiently transfected with vector, PAR2-GFP or PAR2-C361A-GFP. MFI values from 3 independent experiments were used to calculate fold change of cell surface PAR2 and PAR2-C361A relative to vector transfected cells and are displayed as Mean ± SD. **, *P*<0.05; ***, *P*<0.001.

To examine whether the reduced signalling efficiency of PAR2-C361A is due to a reduction in the level of PAR2 expressed on the plasma membrane, we analysed the localization of wildtype and mutant PAR2 expressed by CHO cells. Confocal microscopy was performed on CHO cells transiently co-transfected with wildtype PAR2-mCherry and PAR2-GFP or wildtype PAR2-mCherry and PAR2-C361A-GFP. As indicated by the yellow signal from the merged images shown in Figure 3A, wildtype PAR2-GFP (green) and PAR2-mCherry (red) co-localised throughout subcellular compartments and the plasma membrane. In contrast, PAR2-C361A-GFP (green) co-localised with wildtype PAR2-mCherry (red) in large perinuclear compartments (yellow in merged image) but not at the cell surface where signal was predominantly from wildtype PAR2 (Fig. 3B, red signal in merged image). These data contrast with the findings of Botham and co-workers who showed by flow cytometry that PAR2-C361A is expressed at higher levels than PAR2 on the surface of stably expressing by CHO-Pro5 cells. However, our qualitative data from confocal microscopy analysis were confirmed by quantitative analysis using flow cytometry and cell surface biotinylation assays. As shown in Figure 4A, flow cytometry analysis using anti-PAR2 antibody N19 indicated that PAR2-C361A levels on the cell surface were ∼70% lower than wildtype PAR2 levels. The effect of C361 mutation on cell surface expression of PAR2 was about the same as 2-brompalmitate inhibition of palmitoylation of PAR2 (Fig. 4A). We obtained consistent data by examining plasma membrane expression of PAR2 by cell surface biotinylation. As shown in Figure 4B (left), anti-myc Western blot analysis of intracellular (IC) and plasma membrane (PM) fractions collected from cell surface biotinylated CHO-vector, CHO-PAR2-myc and CHO-PAR2-C361A-myc cells showed much lower levels of PAR2-C361-myc in the PM fraction compared with PAR2-myc. Graphical representation of signals obtained from 3 independent experiments showed that cell surface levels of PAR2-C361-myc were 71.6±4.4% lower than PAR2-myc (Fig. 4B, right). To examine if palmitoylation of PAR2 C361 is required for efficient cell surface expression of PAR2 in an endogenous expressing cells, we transiently transfected DU145 cells with expression constructs encoding wild type PAR2 or palmitoylation deficient PAR2-C361A and followed surface expression by flow cytometry. For this experiment we used anti-PAR2 antibody N19 that will detect both endogenous PAR2 as well as transiently expressed PAR2-myc. As shown in Figure 4C, after subtracting the level of endogenous cell surface PAR2 (identified from DU145-vector cells), DU145 cells transiently expressing C361 PAR2 exhibited 63.9±1.4% less cell surface PAR2 compared with cells transiently expressing wild type receptor. Together these data indicate that palmitoylation of PAR2 at C361 is required for efficient plasma membrane localization of this receptor.

### PAR2 agonism stimulates palmitate incorporation which occurs during secretory trafficking in pre-medial Golgi vesicles

**Figure 5:**
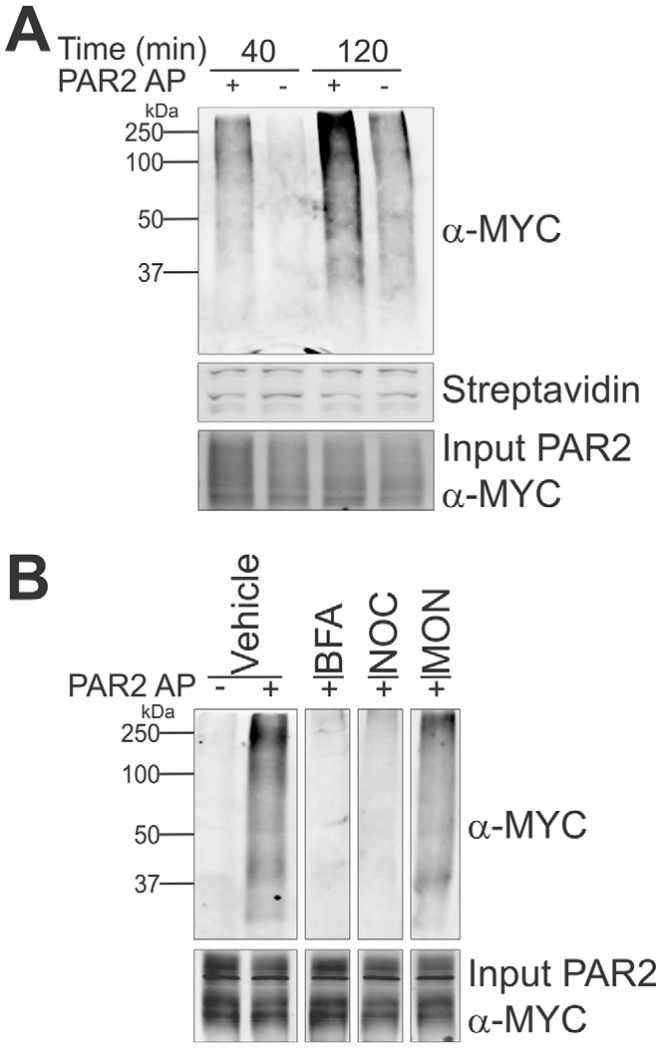
PAR2 agonism stimulates palmitate incorporation which occurs during secretory trafficking in pre-medial Golgi vesicles. *A*, CHO-PAR2-myc cells preincubated with cerulenin for 1 h were labelled with 17-ODYA for 1.5 h in the presence or absence of PAR2-AP (100 µM) for the indicated times. Membrane preparations were collected and biotin-azide reacted to 17-ODYA labelled proteins via click chemistry. Biotinylated proteins were isolated using streptavidin beads. PAR2 palmitoylation was assessed by anti-myc Western blot analysis of bead elutes. *B*, CHO-PAR2-myc cells preincubated with cerulenin were labelled with 17-ODYA for 2 h in the presence or absence of PAR2 AP (100 µM). Nocodazole (20 µg/mL; NOC) was added to medium 15 min prior to labelling with 17-ODYA and monensin (10 µM; MON) and brefeldin A (5 µg/mL; BFA) were added to medium 30 min prior to labelling with 17-ODYA. Labelled proteins were reacted to biotin-azide via click chemistry and biotinylated proteins isolated using streptavidin beads. Palmitoylated PAR2 was detected by anti-Myc Western blot analysis of bead elutes. Data shown in *B* is from cropped lanes from a single Western blot analysis. The data are representative of three independent experiments.

To understand mechanisms resulting in the reduced signalling efficiency and cell surface expression of PAR2-C361A-myc, we examined the effect of agonist stimulation (PAR2 AP SLIGRL-NH_2_) on palmitoylation of wildtype and C361A PAR2 and the intracellular location where palmitoylation occurs. We used metabolic labelling followed by click chemistry mediated biotin labelling of proteins, as described above, to examine the effect of agonism on PAR2 palmitoylation. Membrane fractions were collected from cells metabolically labelled with 17-ODYA in the presence or absence of AP for 40 and 120 min. As shown in Fig. 5A, anti-myc Western blot analysis indicated that incorporation of palmitate in untreated cells increased moderately between 40 and 120 min whereas PAR2 AP caused a significantly larger increase in palmitoylation over this period. These data suggest that PAR2 palmitoylation is induced by receptor agonism.

To examine the subcellular site at which PAR2 palmitoylation occurs, CHO-PAR2-myc cells were labelled with 17-ODYA for 2 h in the presence or absence of PAR2 AP and three different drugs known to reduce protein trafficking through the endoplasmic reticulum (ER) and Gogli organelles: brefeldin A treatment collapses the Golgi apparatus leading to protein accumulation in the ER [36], nocodazole inhibits microtubule formation preventing ER to Golgi transport, leading to protein accumulation in late ER/early Golgi vesicles [37] and monensin impedes protein trafficking leading to accumulation in the medial-Golgi apparatus [36]. Biotin-azide was reacted with equal amounts of 17-ODYA labelled membrane proteins via click chemistry and biotinylated proteins were isolated using streptavidin beads and PAR2 palmitoylation examined by anti-myc Western blot analysis from bead elutes. As shown in Figure 5B (vehicle), agonism induced robust PAR2 palmitoylation in vehicle treated cells and this was blocked in cells treated with brefeldin A (BFA) and nocodazole (NOC) indicating that palmitoylation of this receptor occurs after the ER. In contrast, PAR2 palmitoylation was still apparent in monensin treated cells (MON) indicating that this receptor is palmitoylated in pre-medial Golgi vesicles. These data suggest that PAR2 is palmitoylated in response to agonism during transport through sub-compartments of the Golgi apparatus prior to receptor transport to the cell surface.

### Palmitoylation of PAR2 is required for efficient rab11a-mediated receptor repopulation of the cell surface in response to agonist stimulation

**Figure 6:**
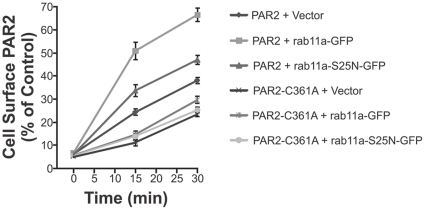
Palmitoylation of PAR2 is required for efficient rab11a-mediated repopulation of the cell surface in response to agonist stimulation. CHO-PAR2-myc and CHO-PAR2-C361A-myc cells transiently transfected with vector, rab11a-GFP or dominant negative rab11a-S25N-GFP were treated with 50 nM trypsin for 15 min to remove cell surface PAR2. Cells were then washed and incubated with trypsin-free DMEM for the indicated times to allow repopulation of PAR2 at the cell surface. Plasma membrane PAR2 was measured by flow cytometry using the anti-PAR2 N19 antibody. MFI values were used to calculate percentage of cell surface PAR2 relative to vector cells. Experiments were performed in triplicate on 3 independent occassions. Data are displayed as mean ± SD. Statistical significant differences - 30 min: PAR2+vector 36.8±1.6%, PAR2+rab11a-GFP 65.5%, *P*<0.0001; PAR2-C361A+vector 22.7±3.5%, PAR2-C361A+rab11a-GFP 33.4±4.0%, *P*<0.05; PAR2-C361A+rab11a-GFP 33.4±4.0%, PAR2+rab11a-GFP 65.5%, *P*<0.0001, compared with PAR2-C361A+rab11a-GFP.

Rab11a, a guanosine triphosphatase (GTPase), regulates exocytic membrane traffic and fusion [45], [46] and enhances PAR2 trafficking to the cell surface [19]. To examine whether palmitoylation is required for agonist stimulated rab11a mediated PAR2 transport to the plasma membrane, we performed antibody N19 flow cytometry analysis of CHO-PAR2-myc and PAR2-C361A-myc cells. These cells were transiently transfected with vector, rab11a-GFP or dominant negative mutant rab11a-S25N-GFP and treated in suspension with trypsin (50 nM) for 15 min before removal of trypsin and monitoring of cell surface expressed PAR2 after 15 and 30 min. As shown in Figure 6, levels of cell surface PAR2-myc in vector transfected cells steadily increased in response to trypsin agonism, indicating that the cell surface was being replenished with nascent receptor. Consistent with previous reports [19], wild type rab11a significantly increased cell surface localization of PAR2 and this effect was slowed by dominant negative rab11a-S25N. Consistent with data in Figure 3 and 4, PAR2-C361A-myc trafficked to the cell surface but at a much lower rate that wildtype receptor (Fig. 6, PAR2-C361A+Vector). In addition, replenishment of the cell surface with this mutant receptor was almost unaffected by rab11a-S25N. However, we note that rab11a induced a statistically significant increase (*P*<0.05) in cell surface PAR2-C361A-myc in response to receptor agonism (Fig. 6). These data indicate that rab11a-mediated trafficking of PAR2 to the cell surface in response to agonism is largely palmitoylation dependent and that there is a smaller, but appreciable, palmitoylation independent component.

### Mutation of PAR2 C361 alters agonist-induced recruitment of β-arrestin-1 and β-arrestin-2

**Figure 7:**
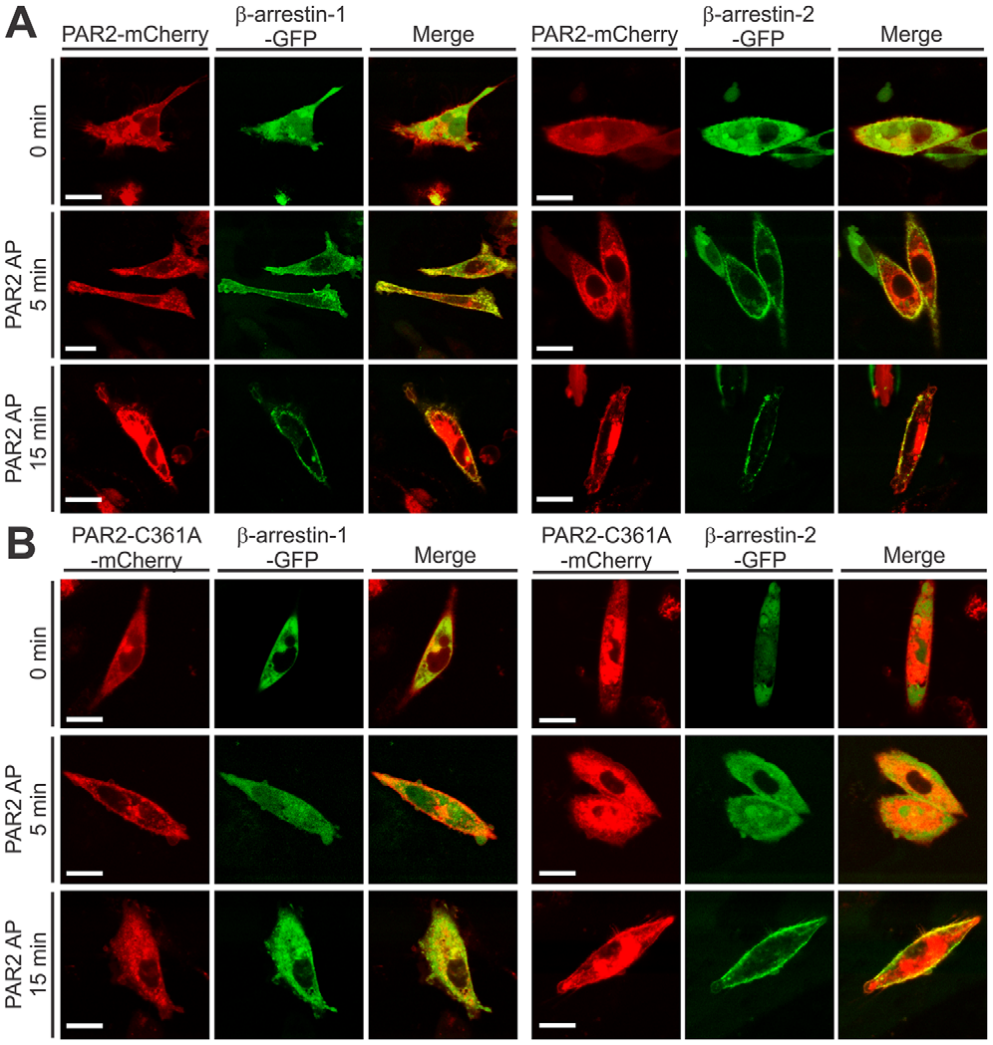
Mutation of PAR2 C361 alters agonist-induced recruitment of β-arrestin-1 delays β-arrestin-2. Confocal microscopy analysis of untreated live cells and at 5 and 15 min after agonism with PAR2 AP (100 µM). *A*, CHO cells transiently co-expressing PAR2-mCherry (red) and β-arrestin-1-GFP (green) or β-arrestin-2-GFP (green). The merge image highlights colocalization (yellow) of PAR2 and β-arrestins. *B*, CHO cells transiently co-expressing PAR2-C361A-mCherry (red) and β-arrestin-1-GFP (green) or β-arrestin-2-GFP (green). The merge image highlights colocalization (yellow) of PAR2 and β-arrestins. Cells were analysed using a Zeiss LSM510 confocal microscope (63× oil immersion objective lens) and images were processed using MetaMorph software and displayed using CorelDraw X5. Images of cells are representative of three independent experiments. *Scale bar*, 10 µM.

β-arrestin-1 and -2 are rapidly recruited to the carboxyl terminus of PAR2 following receptor activation, leading to rapid internalisation [14], [47]. To assess the impact of PAR2 palmitoylation on receptor desensitisation, we examined PAR2 agonist-induced β-arrestin-1 and -2 recruitment by confocal microscopy. CHO cells were transiently co-transfected with β-arrestin-1-GFP or β-arrestin-2-GFP with wildtype PAR2-mCherry or PAR2-C361A-mCherry. As shown in Figure 7A, β-arrestin-1-GFP and β-arrestin-2-GFP localise throughout the cytoplasm of untreated cells co-transfected with either wild type or palmitoylation-deficient PAR2-mCherry. Stimulation of wild type PAR2 with AP for 5 min induced rapid translocation of β-arrestin-1-GFP and β-arrestin-2-GFP to the plasma membrane and colocalization with PAR2 that was sustained for at least 15 min (Fig. 7A, yellow in merged images). In contrast, β-arrestin-1-GFP remained localised to the cytoplasm of cells expressing PAR2-C361A-mCherry under agonist-induced conditions at both time points (Fig. 7B, left). However, agonist stimulation of PAR2-C361A-myc induced a delayed relocation of β-arrestin-2-GFP to the plasma membrane (Fig. 7B, right, yellow in merged image at 15 min). These data suggest that PAR2 palmitoylation is essential for agonist induced relocation of β-arrestin-1 to the cell surface but not for relocation of β-arrestin-2.

### PAR2 C361 is required for efficient agonist-induced receptor endocytosis and degradation

**Figure 8:**
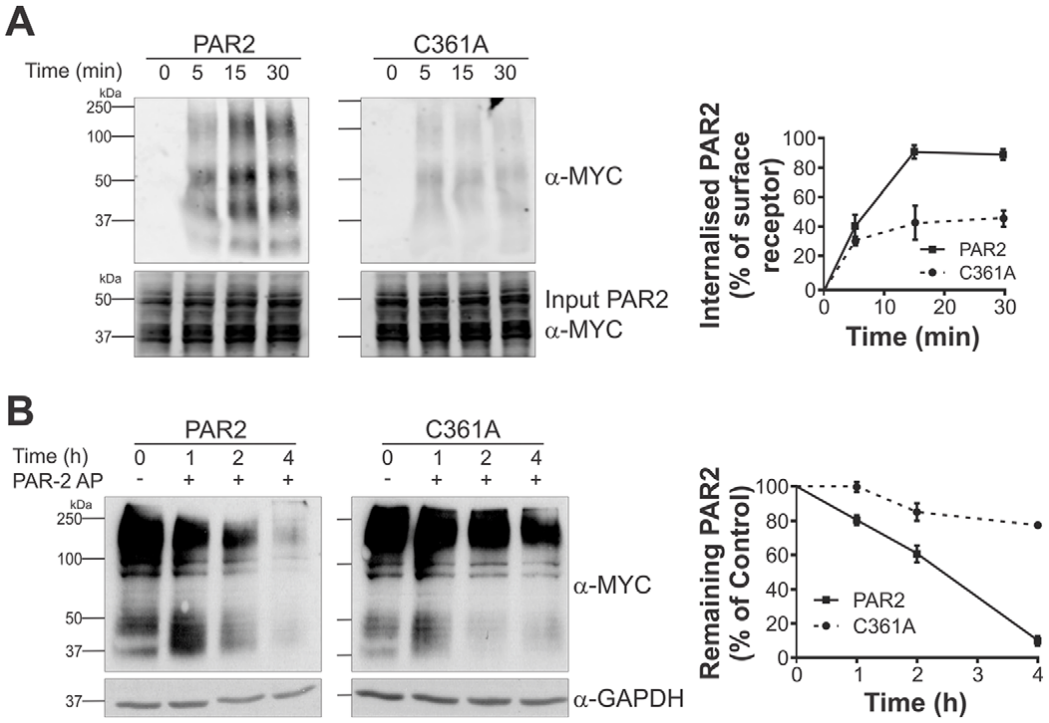
PAR2 C361 is required for efficient agonist-induced receptor endocytosis and degradation. *A*, CHO-PAR2-myc and CHO-PAR2-C361A-myc cells labelled with membrane impermeant EZ-link NHS-SS-biotin were treated with PAR2 AP (100 µM) for the indicated times to induce receptor internalisation. Protein degradation was blocked by incubation of cells with the proteasome inhibitor MG132. Residual cell surface biotin was removed by washing with MeSNA and internalised biotin-labelled protein was isolated from whole cell lysates using streptavidin beads. Anti-myc Western blot analysis was performed on bead eluates and input lysates. Data are representative of 3 independent experiments with graphical representation of densitometry analysis of these data shown in the right panel. Percentage of internalised receptor was calculated by dividing the value for internalised PAR2 at each time point by the value for total surface PAR2 which was determined from an experiment performed in parallel in which cells were not treated with AP or MeSNA. Values are displayed as Mean ± SD. *B*, CHO-PAR2-myc and CHO-PAR2-C361A-myc cells were treated with PAR2 AP (100 µM) for the indicated times in the presence of 70 µM cycloheximide, to prevent *de novo* protein synthesis. Lysates were examined by anti-myc and anti-GAPDH Western blot analysis to assess the levels of PAR2 remaining after receptor agonism. Data are representative of 3 independent experiments with graphical representation of densitometry analysis of these data shown in the right panel. Values are displayed as Mean ± SD. PAR2 endocytosis and degradation were significant compared with PAR2-C361A; *P*<0.001.

Our data indicate that compared with wildtype receptor, PAR2-C361A displays reduced trafficking to the cell surface, lower levels of plasma membrane localization and diminished β-arrestin recruitment following receptor agonism. As it has previously been reported that after β-arrestin recruitment, agonism of PAR2 results in rapid receptor internalisation and sorting for lysosomal degradation [14]–[46], we evaluated the effect of loss of palmitoylation on the rate of endocytosis and degradation of this receptor. To assess endocytosis, we examined the rate of agonist-induced internalisation of wildtype and C361A PAR2. CHO cells stably expressing wildtype PAR2-myc or PAR2-C361A-myc labelled with biotin were treated with PAR2 AP (100 µM) for 5, 15 and 30 min to allow endocytosis. After these periods of internalisation, residual cell surface biotin was removed by washing cells with MeSNA. Endocytosed proteins labelled with biotin were isolated from whole cell lysates using streptavidin beads and the level of. PAR2 endocytosis examined from bead elutes by anti-myc Western blot analysis. As shown in Figure 8A, PAR2-myc endocytosis steadily increased over 30 min in response to PAR2 AP with the internalised receptor detected following 5 min and maximal PAR2 endocytosis detected at 15 min. In contrast, under the same conditions, internalisation of PAR2-C361A-myc was greatly reduced (Fig. 8A). Graphical analysis of densitometric analysis of three independent Western blot analyses normalised to total cell surface PAR2 indicated that mutation of C361 reduced the level of PAR2 internalisation by approximately 50% (Fig. 8A). These data indicate that palmitoylation is required for efficient agonist-induced PAR2 endocytosis.

To assess receptor degradation CHO-PAR2-myc and CHO-PAR2-C361A-myc cells, treated with cycloheximide to inhibit *de novo* protein synthesis, were incubated with PAR2 AP. As shown in Figure 8A, anti-myc Western blot analysis indicated that PAR2-myc levels dropped markedly over a 4 h period following AP treatment. In contrast, under the same conditions the reduction in PAR2-C361A-myc levels was much less pronounced (Fig. 8 B). Graphical analysis of densitometric analysis of three independent Western blot analyses indicated that 4 hours after AP treatment levels of PAR2-myc dropped by 90±4.5% whereas PAR2-C361A-myc reduced by 22.6±2.4% (*P*<0.0001, compared with PAR2-myc) (Fig. 8B). These data indicate that loss of palmitoylation reduces the rate of degradation of PAR2 in response to agonist stimulation.

## Discussion

We have examined the role of palmitoylation in trafficking to and from the cell surface of the protease activated GPCR PAR2 as well as the effect of this post-translational modification on receptor signalling. Our data extend findings from a recent report by Botham and colleagues [21] who demonstrated that palmitoylation of PAR2 can occur at C361. However, in several respects our data are also inconsistent with a number of the key results of this group. We show for the first time that inhibition of palmitoylation reduces the cell surface localization of PAR2 by at least 50% in endogenous expressing cell lines (PC3, DU145 and 22Rv1). We also show that PAR2-C361A transiently expressed by one of these endogenous expressing cells lines (DU145) is inefficiently trafficked to the cell surface compared with wildtype PAR2. These data suggest that palmitoylation is required for efficient plasma membrane expression of endogenously expressed PAR2. This conclusion is the converse of the findings of Botham *et al.* who showed by flow cytometry that palmitoylation-deficient PAR2, stably expressed by CHO-Pro5 cells, is present on the plasma membrane at between ∼150% (70% confluence) and ∼175% (20% confluence) of the levels of wildtype PAR2 [21]. However, our data are supported by flow cytometry and cell surface biotinylation studies of wildtype and C361A PAR2 stably expressed by CHO-K1 cells as well as of confocal microscopy analysis of transiently transfected cells. In addition to demonstrating that PAR2 is exclusively palmitoylated at C361, these approaches showed, consistent with our observations from endogenous expressing cells, that mutation of C361 results in at least a 60% reduction in the cell surface expression of PAR2.

**Figure 9:**
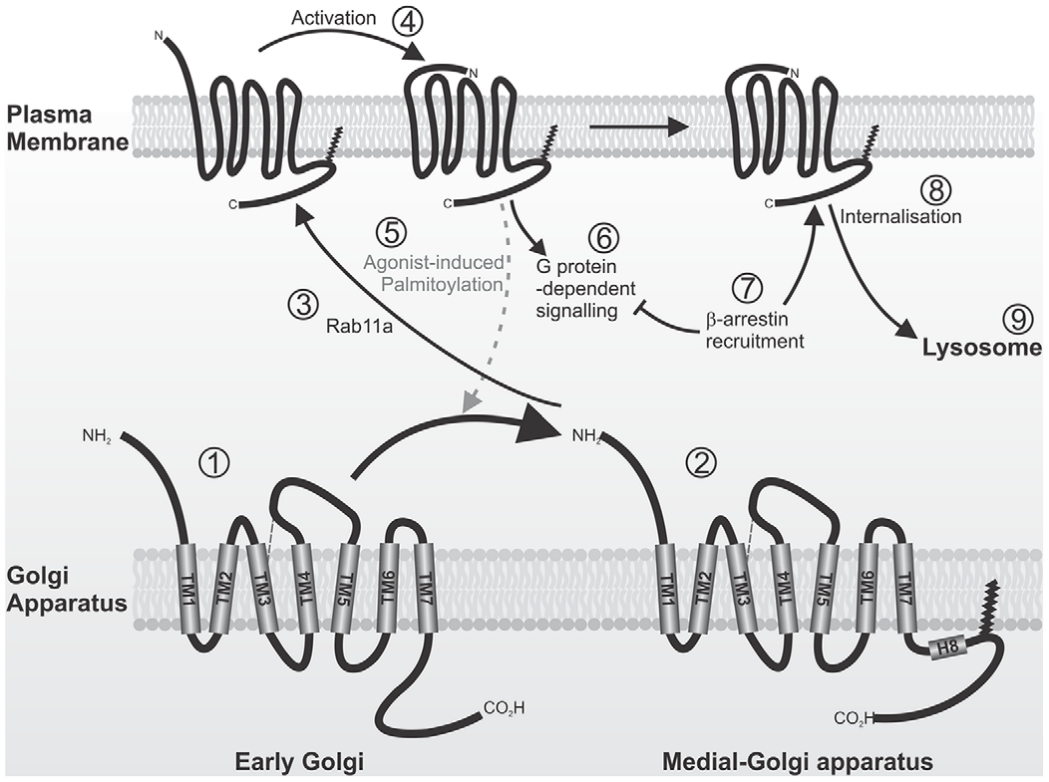
PAR2 palmitoylation is required for optimal receptor signalling, cellular trafficking and plasma membrane expression. Unpalmitoylated PAR2 located in Golgi vesicles (1). Palmitoylation occurs within the cis- to medial-Golgi (2). This anchors the carboxyl domain of PAR2 to the inner leaflet of cell membranes likely resulting in the formation of an intracellular eighth α-helix (H8) required for effective signalling (2). Interactions of palmitoylated PAR2 with the GTPase rab11a promotes agonist induced receptor repopulation of the cell surface (3). Activation of PAR2 (4) enhances palmitoylation of Golgi localised PAR2 (5) and, reciprocally, receptor palmitoylation is required for efficient PAR2 signal transduction (6). Receptor agonism induces β-arrestin translocation to the plasma membrane (7) which mediates non-G protein signal transduction and receptor internalisation (8). Internalised PAR2 is trafficked and sorted for lysosomal degradation via early and late endosomes (9).

As depicted in Figure 9, our data indicate that palmitoylation of PAR2 is required for optimal receptor signalling, cellular trafficking and plasma membrane expression. We propose that mono-palmitoylation of PAR2 at C361 occurs in the Golgi apparatus prior to transit to the *medial*-Golgi and this is enhanced by receptor agonism at the cell surface. Efficient cell surface repopulation of PAR2 by the GTPase rab11a is also dependent on receptor palmitoylation and in its absence, nascent PAR2 accumulates in pre-Golgi vesicles or the early Golgi, preventing efficient trafficking toward the plasma membrane. Our calcium flux data indicate that palmitoylation is also required for efficient PAR2 signalling via Gα_q_. However, we note that the observed reduction in Gα_q_ signalling via PAR2-C361A compared with wildtype receptor is likely largely due to the reduced expression of PAR2 on the cell surface. It has previously been shown that receptor activation induces translocation of β-arrestin from the cytosol to the plasma membrane to facilitate non-G protein mediated signal transduction and PAR2 internalisation [14], [47]. However, agonism of cells expressing palmitoylation-deficient PAR2 fails to induce β-arrestin-1 translocation while β-arrestin-2 translocation is delayed. As our confocal, flow cytometry and cell surface biotinylation assays indicated that palmitoylation deficient PAR2 is capable of trafficking to the plasma membrane, albeit with reduced efficiency compared with wildtype receptor, the observed effects on β-arrestins suggest that palmitoylation is required for efficient internalisation of PAR2. This was supported by assays showing that endocytosis of this receptor is markedly reduced when C361 is mutated. Consistently, degradation of PAR2-C361A following receptor agonism occurs at much lower levels than wildtype PAR2, likely because lower levels of the mutated receptor are trafficked to the cell surface and available for activation. Thus, our model suggests that palmitoylation of PAR2, induced by cell surface agonism, is required for efficient trafficking from Golgi stores to the plasma membrane. At this location, palmitoylation is also required for efficient recruitment of proteins that are needed for signal transduction, receptor desensitisation and internalisation and sorting for lysosomal degradation. Accordingly, these data suggest that palmitoylation is an essential post-translational modification in the lifecycle of PAR2.

Our finding that palmitoylation of PAR2 occurs following ER export and during Golgi transit is consistent with the observation that 12 of the 23 known human PATs are located within the Golgi apparatus [48], [49]. In addition, several other proteins are palmitoylated at this site including endothelial nitric oxide synthase [49], tetraspanins CD9 and CD151 [50], [51], [52]. Furthermore, several GPCRs are palmitoylated during transport from ER-Golgi intermediate compartments through to the medial Golgi including CCR5 [53], [54], δ-opioid receptor [55], thyrotropin receptor [56] and vasopressin V1a receptor [57]. Further work is required to identify the PAT(s) that palmitoylate PAR2 within the Golgi apparatus. In addition, based on the diverse localization of PATs within cells and the dynamic nature of *S*-palmitoylation, it is possible that PAR2 will be palmitoylated at other cellular locations as occurs for the δ-opioid receptor [55] and the β_1_-adrenergic receptor [58].

The palmitoylation of Golgi localised PAR2 in response to receptor agonism and prior to cell surface expression, suggests that this modification facilitates plasma membrane targeting. In support of this, the palmitoylation inhibitor 2-bromopalmitate reduced cell surface expression of both endogenous and over-expressed PAR2. Moreover, PAR2-C361A, although capable of trafficking to the cell surface, displayed greatly reduced plasma membrane localization. Consistent with these observations, other GPCRs and membrane receptors also display reduced cell surface expression following chemical inhibition of palmitoylation or mutation of carboxyl terminal palmitoylation sites. These include the chemokine CCR5 receptor [53], [54], dopamine D1 receptor [59], follicle-stimulating hormone receptor [60], histamine H2 [61], lutropin/choriogonadotropin receptor [62], δ-opioid receptor [55], V2R [41], [63]. In addition, we note that there are examples of GPCRs (V1a vasopressin receptor, β_1_R and β2R) that show unaffected cell surface expression when palmitoylation is blocked [42], [57], [58]. Accordingly, the proposal by Botham and colleagues that mutation of the PAR2 palmitoylation site results in elevated cell surface expression [21] should be viewed with caution. It is not clear why the data of these workers differ from our own findings, although it is possible that use of different CHO cell sub-lines may be a contributing factor. Whereas we used CHO-K1 cells, Botham and colleagues employed the CHO-Pro5 line which is the parental cell line for several glycosylation defective sub-lines and an auxotroph requiring proline supplementation [64].

Several years ago the crystal structure of the class A GPCR bovine rhodopsin revealed that a partially exposed α-helix, designated α-helix 8 (α-H8), is located between the final transmembrane domain and the palmitoylated cysteines in the carboxyl terminus of this receptor [65]. Since then this domain has been identified in other GPCRs containing consensus carboxyl terminal palmitoylation sites including cannabinoid receptor 2, β2R, adrenoreceptor [66], cannabinoid receptor 1 [67] and β1-adrenergic receptor [26]. It is clear from our study and the report of Botham *et al.* [21] that C361 of PAR2 is palmitoylated and this modification is at the carboxyl boundary of a putative PAR2 α-H8. These domains have been shown to be required for direct coupling of several GPCRs with G protein subunits [28], [66], [68], [69], [70], and it is possible the α-H8 region functions by switches between inactive (helical) and active (non-helical) in response to ligand binding. Very recently the α-H8 of PAR1 was shown to be critical for G_q_-dependent receptor signalling [28] and the direct relevance of this domain to PAR2 signalling is suggested by the observation that its deletion completely prevents Gα_q_ signal transduction [71]. Accordingly, it is possible that like other GPCRs, PAR2 palmitoylation contributes to α-H8 stability thereby enabling efficient G protein coupling. In addition, it is possible that PAR2 palmitoylation-induced stabilization of its α-H8 is required for rab11a-mediated receptor repopulation of the cell surface in response to agonist stimulation. This is based on the observation that palmitoylation stabilises the α-H8 of the prostacyclin receptor and this is required for its interactions with rab11a during receptor internalization and recycling [72]. Consistently, we observed that whereas agonist-induced cell surface localization of wildtype PAR2 was significantly increased by rab11a, replenishment of the cell surface with mutant receptor was almost unaffected by this GTPase. Thus, although we did not address this possibility experimentally, the PAR2 α-H8 may mediate any direct interactions between receptor and rab11a allowing efficient transport to the plasma membrane.

Our results indicate that mutation of the PAR2 palmitoylation site also negatively impacts on agonist-induced β-arrestin colocalization with this receptor at the plasma membrane. Similar effects on β-arrestin recruitment have been reported for the GPCRs V2R [73] and TRH receptor [74]. Interestingly, palmitoylation has been shown to alter phosphorylation of the GPCRs 5-hydroxytryptamine_4a_ receptor [75], β2R [42], CCR5 [76], TRH receptor [77] and vasopressin V1a receptor [57] and of the AMPA membrane receptor GluR1 [78]. Accordingly, as it is known that phosphorylation of the PAR2 carboxyl tail is a key regulator of β-arrestin recruitment to this receptor [15], we speculate that palmitoylation is a necessary prerequisite for the phosphorylation that is essential for β-arrestin recruitment to PAR2.

In summary, these data provide new insights on the role of palmitoylation in the life cycle of PAR2. The data indicate that this post-translational modification is required for maximal cell surface expression of endogenous and stably expressed PAR2 and is needed for both efficient signalling and agonist-induced rab11a-mediated PAR2 trafficking to the plasma membrane. At the plasma membrane agonist-induced β-arrestin recruitment is compromised by mutation of the PAR2 palmitoylation site as is receptor degradation in response to agonism. We also note that loss of palmitoylation does not completely block these PAR2 cellular processing events. Accordingly, further studies are required to examine other mechanisms regulating this receptor.
